# CC Chemokine Receptor 5: The Interface of Host Immunity and Cancer

**DOI:** 10.1155/2014/126954

**Published:** 2014-01-19

**Authors:** Carlos Eduardo Coral de Oliveira, Julie Massayo Maeda Oda, Roberta Losi Guembarovski, Karen Brajão de Oliveira, Carolina Batista Ariza, Jamil Soni Neto, Bruna Karina Banin Hirata, Maria Angelica Ehara Watanabe

**Affiliations:** Laboratory of Polymorphism and Application Study of DNA, Department of Pathological Sciences, Biological Sciences Center, State University of Londrina, Celso Garcia Cid highway, Pr 445, Km 380, 86057-970 Londrina, PR, Brazil

## Abstract

Solid tumors are embedded in a stromal microenvironment consisting of immune cells, such as macrophages and lymphocytes, as well as nonimmune cells, such as endothelial cells and fibroblasts. Chemokines are a type of small secreted chemotactic cytokine and together with their receptors play key roles in the immune defense. Critically, they regulate cancer cellular migration and also contribute to their proliferation and survival. The CCR5 chemokine receptor is involved in leucocytes chemotaxis to sites of inflammation and plays an important role in the macrophages, T cells, and monocytes recruitment. Additionally, CCR5 may have an indirect effect on cancer progression by controlling the antitumor immune response, since it has been demonstrated that its expression could promote tumor growth and contribute to tumor metastasis, in different types of malignant tumors. Furthermore, it was demonstrated that a CCR5 antagonist may inhibit tumor growth, consisting of a possible therapeutic target. In this context, the present review focuses on the establishment of CCR5 within the interface of host immunity, tumor microenvironment, and its potential as a targeting to immunotherapy.

## 1. Introduction

Chemokines are a type of small secreted chemotactic cytokine. Together with their receptors, they play key roles in the immune defense by directing and controlling the migration, activation, differentiation, and survival in the physiology of acute and chronic inflammatory processes as well as in the pathological deregulations by attracting and simulating the various subsets of specific leukocytes [1]. Moreover, chemokines critically regulate cancer cellular migration and also contribute to their proliferation and survival [2].

The identification of a large number of chemokine receptors and their selectivity characterization and expression have provided information on the traffic regulation of leukocytes in health and disease. They are expressed on different types of leukocytes constitutively or induced, depending on cell types [3].

The chemokine system is often thought as showing significant redundancy since one receptor can bind multiple ligands, and conversely, a single ligand can bind several chemokine receptors [4]. However, differential spatio-temporal expression patterns for different chemokines and receptors in our body indicate that they probably have distinct roles *in vivo *[5]. To date, about 45 chemokines and 20 chemokine receptors have been identified and are grouped into four categories (C, CC, CXC, and CX3C) based on the location of the main cysteine residues near the N-termini [6]. Chemokine receptors relay their signal through heterotrimeric G-proteins [7].

The CC chemokine receptor 5 (CCR5) belongs to the trimeric guanine nucleotide-binding-protein-coupled seven-transmembrane receptor superfamily, which comprises the largest superfamily of proteins in the body [8]; exerts its activity via G protein; and binds to the chemokines RANTES (CCL5), MIP-1*α* (CCL3), and MIP-1*β* (CCL4) [9]. This receptor is involved in the chemotaxis of leucocytes to inflammation sites [10] and plays important role in the recruitment of macrophages, T cells, and monocytes in inflammation [11].

A common 32-base pair deletion mutation (delta32) in the CCR5 gene causes truncation and loss of CCR5 on lymphoid cell surfaces of homozygotes. The delta 32 deletion typically results in complete retention of CCR5 in the endoplasmic reticulum within homozygous or diminished CCR5 expression in heterozygous on the cell membrane [12]. Also, it is interesting that the CCR5 expression is under the control of a complexly organized promoter region upstream of the gene. The main transcriptional activity of the CCR5 promoter region is contained within the downstream promoter P1, which is transactivated by the transcription factor cAMP responsive element binding protein 1 (CREB-1) [13]. Study from Wierda et al. [14] reveals that epigenetic mechanisms involving DNA methylation, histone acetylation, and methylation modifications contribute to the transcriptional regulation of CCR5 expression.

It is known that stromal microenvironment may play active roles in cancer pathogenesis with the participation of chemokine receptor CCR5. Although cancer tissue consists of various stromal cells, such as leukocytes, fibroblasts, and endothelial cells, there is little known about the driving forces of cells migration and infiltration into cancer tissue. In this context, the present review focuses on the establishment of CCR5 receptor within the interface of host immunity, tumor microenvironment, relation with T regulatory (Treg) cells in cancer, and also its potential as a targeting to immunotherapy.

## 2. CCR5 Receptor and the Interface of Host Immunity

Chemokines present the potential to stimulate T-cell activation, although the pattern of activation may differ for different chemokine-chemokine receptor interactions [15].

Expression of chemokine receptors can define subtypes of T lymphocytes. Mature peripheral T lymphocytes express different chemokine receptors' profiles depending on their functional phenotype. For example, T-helper lymphocyte type 1 (Th1), which synthesize interleukin-2, interferon-*γ* and mediate phagocyte activation, express CXCR3 (chemokine (C-X-C motif) receptor 3), CCR2 (C–C chemokine receptor type 2) and CCR5. On the other hand, T-helper lymphocytes type 2 (Th2) produce interleukin-4 and interleukin-5, which mediate B lymphocyte antibody production and express CCR3, CCR4, and CCR2. These differences partly determine the type of immune response that will be deployed at an inflammation site [16, 17]. In this context, CCR5 regulates trafficking of lymphoid cells such as memory/effector Th1 lymphocytes or myeloid lineage cells (e.g., monocytes, macrophages, and immature dendritic cells) and microglia [18].

During recent years, it has become evident that a subpopulation of T cells named T regulatory cells (Tregs) plays a major role in sustaining tolerance to self-antigens. Interesting expression of CCR5 was detected on Tregs [19] and CD103+ effector/memory Tregs could express CCR5 [20]. Newer fates for helper T cells continue to be identified, with differentiation based on production of their signature cytokines and master regulator transcription factors, such as Th9, Th17, and Th22 cells [21, 22].

The importance of CCR5 for a proper immune response is very much dependent on the type of stimuli; moreover, in some cases, compensating mechanisms override the absence of CCR5 expression and function. It has been suggested that CCR5 has a far more important role in the immune response than in regulating the trafficking of immune cells [23].

CCR5 was found to possess various functions, other than chemotaxis. Activation of these receptors can induce a costimulatory effect and IL-2 secretion by T cells and IL-12 secretion by macrophages [24, 25] and serves as antiapoptotic signals for macrophages under viral infection [26].

The expression of this receptor is markedly upregulated upon T-cell activation, which allows activated T cells to migrate towards sites of inflammation. However, although selective expression of CCR5 and CXCR3 on Th1 cells has been suggested [27], others demonstrated that CCR5 is equally present on all activated T cells independently of their functional Th polarization [16].

Cytokines and chemokines have a crucial role in cancer-related inflammation with consequent, direct, and indirect effects on the proliferative and invasive properties of tumor cells [28].

Thus, in addition to the expression of the chemokine receptor CCR5, expression of ligands of this receptor is responsible for the attraction of lymphocytes to the tumor microenvironment.

CCR5 endogenous ligands include the main chemokines CCL3 (MIP-1*α*), CCL4 (MIP-1*β*), and CCL5 (RANTES). Their order of potency of metabolic activity is CCL3 > CCL4 = CCL5 [9]. Identification of suppressor factors produced by CD8+ cells that counter infection by certain HIV-1 strain infections [29] previewed the critical identification of CCR5 as being one of two chemokine receptor molecules that serve as coreceptors for HIV-1 entry [30].

Macrophages, lymphocytes, and natural killer cells are the predominant cell types of the immune cells in cancer tissue. In addition, eosinophils, granulocytes, and B cells are present as minor immune cells in some cancers [31]. It is known that the chemokine CCL5 is highly expressed in cancer where it contributes to inflammation and malignant progression, but the question of which cancer cell-derived chemokines are friends and which are enemies remains unanswered.

## 3. CCR5 Receptor and the Interface of Cancer

Chemokines are representative driving forces of leukocytes in the inflammatory process [32, 33], leading to the question whether or which chemokines secreted from cancer cells are specifically correlated with cancer progression via infiltration of leukocytes into cancer tissue. It has been postulated that cancer cell-derived chemokines increase the infiltrating immune cells in a particular cancer type and promote or suppress cancer progression according to the type and immune effect potency of the infiltrating cells [6].

Chemokines binding to their receptors can induce a serie of intracellular cascade reactions which may regulate the migration of cancer cells [34]. Some reports indicate that the expression of chemokine receptors in many cancer cells is not random [35] and may play a role in organ-specific metastasis. It has been also demonstrated that tumor cells can create autocrine gradients of chemokine receptors that guide their migration in a luring gradient under the influence of interstitial flow towards functional lymph nodes, even if lymphatic endothelial cells are absent, although the effect is greatly amplified when both flow and cells are present. This process was denominated “autologous chemotaxis,” suggesting that chemokines are secreted by tumor cells themselves [36].

CCR5 may have an indirect effect on cancer progression by controlling the antitumor immune response [37]. Intermediate and strong CCR5 expression were significantly associated with nonmetastatic colorectal cancer and correlated with both the infiltration of tumor margins with CD8+ T-lymphocytes and the absence of (lymphatic) metastasis. Weak or absent CCR5 expression was significantly associated with lymph node metastasis and advanced UICC (Union for International Cancer Control) stages III and IV. It is hypothesized that T-cell retention at the tumor site seems to be mediated by CCR5-dependent mechanisms of the immune and tumor cells. CCR5 might play a role during progression of colorectal carcinoma, possibly opposing to cancer progression. However, CCR5 expression of tumor cells might as well be an epiphenomenon regulated by specific chemokines [38].

CCR5 expression on CD8+ T cells was necessary for their efficient activation and migration to the tumor site and for tumor killing; importantly, CCR5 must also be expressed by CD4+ T lymphocytes to achieve maximal CD8+ T cell effector function [39]. Additionally, CCL5 was demonstrated to promote chemotaxis of monocytes, increasing MMP9 expression in MCF7 cells [40] and angiogenesis, partly dependent on vascular endothelial growth factor (VEGF) secretion by endothelial cells [41], suggesting that the expression of CCL5 by cancer cells results not only in monocyte migration to the tumor site but also in protumorigenic activities of this chemokine and of proinflammatory cytokines that may facilitate metastasis formation and contribute to disease progression.

Metastasis represents the definite cause of 90% of deaths from solid tumors and it emerges from the somatic evolution of a genetically variegated cancer-cell population under the selective pressures of an environment that imposes tight rules on cellular behavior. The complete inefficiency of the metastatic process implies that healthy tissues naturally display a marked hostility toward invading tumor cells, but certain cell lineages may express molecules that bias the metastatic efficiency to different target organs [42].

It has been demonstrated that CCR5 expression could promote tumor growth and contribute to tumor metastasis. van Deventer et al. [43] showed that mice expressing CCR5 present enhanced local tumor growth and an impaired response to vaccine therapy compared to CCR5 knockout mice. The authors showed that CCR5 expression in stromal cells, but not hematopoietic cells, contributed to tumor metastasis. As an example, CCR5 is involved in metastasization of chondrosarcomas [44] and in migration of oral cancer cells [45]. Its expression correlates with the ability of aggressive natural killer cell leukemia cells to infiltrate into multiple organs [46] and with multiple myeloma cell growth, bone marrow homing, and osteolysis [47].

Hodgkin Lymphoma (HL) cell lines, including Reed-Sternberg cells, has been shown to express CCR5, and clonogenic growth of these cells were directly attributed to engagement by different CCR5 ligands. Aldinucci et al. [48] demonstrated that transducing proliferation signals and chemotaxis of eosinophils and CD4+ T lymphocytes into HL-involved tissues were mediated by CCR5/CCL5 and this axis actively contributed to the formation of typical HL cellular microenvironment [48].

The prognosis of patients with osteosarcoma distant metastasis is generally considered as very poor. Wang et al. [49] examined the migratory activity of human osteosarcoma cells through CCL5 gradient. They verified that CCL5-CCR5 interaction increases the expression of *α*v*β*3 integrin via MEK, ERK, p65, and NF-*κ*B dependent pathway, contributing to migration of human osteosarcoma cells.

Lin et al. [50] have verified that CCR5 and CCL5 were highly expressed in breast cancer lymph nodes metastasis. Treatment with the cytokine TNF-*α* increased the expression of CXCR2, CX3CR1, CCR9, and CCR5 in breast cancer MCF-7 cell line and caused a marked increase in the expression of CXCR2 and CCR5. This is also observed in the highly metastatic MDA-MB-231 cell line, although the levels of expression observed after cytokine stimulation are higher than those obtained in the MCF-7 cell line. Basal expression of a given chemokine receptor is not by itself a good marker of homing or aggressiveness and is subject to change by the microenvironment. There are cell subpopulations expressing different levels of chemokine receptors, which, under a particular stimuli, change their expression levels and thus their aggressiveness. Recently, Wang et al. [51] demonstrated that CCR5 and CCL2 serum levels increased during the period from benign change to benign change with proliferation in samples of patients with breast mass, suggesting that they might be involved not only in the malignant process, but also in the benign process before atypia.

Human adipose-derived stem cells represent a cellular source of CCL5 which influences tumor cell migration and invasion of human breast cancer cell line MDA MB 231 in paracrine and autocrine fashion [52]. Bone-marrow-derived mesenchymal stem cells have been found to integrate into the tumor-associated stromal and secrete CCL5 which then acts in a paracrine way on the cancer cells to enhance their invasion [53]. However, Jayasinghe et al. [54] demonstrated that tumor-derived CCL5 expression alone does not make a significant contribution to disease progression and pointed towards a role for host-derived CCL5 in breast cancer.

CCR5 was also expressed in skin biopsies of acute myeloid leukemia. The autocrine production of CCL3 together with CCR5 expression would facilitate the retention of the acute myeloid leukemia blasts in the skin. CCR5/CCL3 and CXCR4/CXCL12 interactions facilitate the retention of acute myeloid leukemia cells in the skin, and CXCR7/CXCL12 interactions subsequently prolong their survival [55].

Notch pathway is a well-known factor in the development of lymphoid lineage [56–58]. Mirandola et al. [59] investigated the possible regulative role of Notch1 in the expression and function of chemokine receptors CCR5, CCR9, and CXCR4 that play a role in determining blast malignant properties and localization of extramedullary infiltrations in leukemia. In this context, these authors suggested that Notch1 pathway abnormalities trigger an increase of CCR5 and CCR9 expression on leukemic blasts.

In last years, it has become evident that a subpopulation of T cells, named T regulatory cells, represents an integral part of the immune system and plays an important role in the interface of tumor and host immunity in breast cancer [60]. Regulatory T cells (CD4+CD25+Foxp3+) are known to be involved in suppressing immune responses [61], and they also express CCR5 on their surface, enhancing migration to the peripheral inflamed tissues [62, 63] and gravid uterus [64] through interaction with CCR5 ligands.

## 4. CCR5 and Treg Cells: The Interface of Cancer

T cells present an important immunological response in tumor growth and they become Tregs in the presence of appropriate stimuli and interactions with tumor cells. These regulatory cells and the transforming growth factor beta (TGF-*β*) are immunological effectors that act in a coordinated manner, once that TGF-*β* stimulates the development of Tregs and also is one of the cytokines produced by them and by the tumor cells themselves, a mechanism that has important implications in tumor progression [65].

Tumors evade immune destruction by actively inducing immune tolerance through the recruitment of CD4+CD25+Foxp3+ regulatory T cells. CD4+Foxp3+ Tregs, compared with CD4+Foxp3-effector T cells, preferentially express CCR5. Treg cells migration into the tumor microenvironment is mediated by the CCL5/CCR5 axis in pancreatic adenocarcinoma, and blockade of this pathway may represent a novel immunomodulatory strategy for the treatment of cancer. Disruption of CCR5 signaling in the tumor slows tumor growth through a Treg-mediated mechanism, although disruption of the CCL5/CCR5 axis skews migration of only about 20% of the CD4+ population (less Tregs, more Teffs). Its physiological importance is reflected in the effects on tumor growth [66]. Moreover, data from Chakraborty et al. [67] which have explored the chemokine receptor expression profile and function showed that *ex vivo* cultured Tregs retained the expression of CCR7 but dramatically downregulated CCR5 as compared with freshly isolated Tregs.

In addition, Chang et al. [20] investigated the antitumor ability of CD103^+^ and CD103^−^ Treg cells *in vivo* and *in vitro.* They found that the potent *in vivo* suppression ability of CD103^+^ Tregs is due to the tissue-migration ability through CCR5 expression.

CD4+ Tregs infiltrate renal cell carcinoma, and their numbers predict poor prognosis [68]. Tregs mediate effector T-cell suppression and thereby could aid tumor immune escape. CCR5, CXCR3, and CXCR6 are involved in the selective recruitment of T cells into renal cell carcinoma tissue. Thus, these chemokine receptors, along with CCR6, are involved in recruiting Tregs to the tumor site. Reducing Treg recruitment by blocking CCR5-, CXCR3-, and CXCR6-mediated homing risks prevents recruitment of naturally occurring antitumor T cells [69].

Chang et al. [70] found that higher levels of CCL5 expression in human and murine colon tumor cells correlated with higher levels of CD8+ T cells apoptosis and infiltration of Tregs. In this study, TGF-*β* signaling blockade diminished apoptosis of CD8+ T cells, implicating TGF-*β* as an effector of CCL5 action. Their findings establish that CCL5/CCR5 signaling recruits Tregs to tumors and enhances their ability to kill antitumor CD8+ T cells, thereby defining a novel mechanism of immune escape in colorectal cancer.

Tan et al. [66] established that Treg migration to pancreatic adenocarcinoma is driven, at least in part, by CCR5 chemotaxis, and further demonstrated that disruption of CCR5 chemotaxis might be a useful strategy for impairing recruitment of tumor-associated Tregs, thereby slowing tumor growth. Considering this aspect, CCR5 could be a promising target for immunotherapy.

Elucidating the types of cells recruited and signal pathways involved in the crosstalk between tumor cells and stromal cells will help to identify novel strategies for cotargeting cancer cells and tumor stromal cells to suppress metastasis and improve patient outcome [71].

Myeloid-derived suppressor cells (MDSCs) are one of the key suppressor cells that regulate antitumor immune responses in conjunction with Tregs in tumor-bearing hosts [72]. Recently, it was demonstrated that mouse tumor-infiltrating granulocytic and monocytic (MO-MDSC) myeloid-derived suppressor cells expressed increased levels of chemokines comprising the CCR5 ligands CCL3, CCL4, and CCL5, and they were responsible to recruit high numbers of Tregs. Intratumoral injection of CCL4 or CCL5 increased tumor-infiltrating Tregs, and deficiency of CCR5 led to their profound decrease, emphasizing the importance of CCR5 in the control of antitumor immune responses [19].

A chemokine receptor antagonist of the CCL5 receptors, CCR5 and CCR1, was shown to inhibit experimental breast tumor growth, further implicating CCL5 as an important molecule in breast cancer [73]. Regarding this aspect, Velasco-Velazquez and Pestell [74] used preclinical models and demonstrated that CCR5 promotes basal breast cancer subtype invasiveness and metastatic potential, while CCR5 inhibition abrogates them [75].

Understanding cellular and molecular mechanisms of tumor pathogenesis is critically important for the development of new approaches to cancer treatment. Many studies showed that cellular structures and receptors play an essential role in molecular and physiological processes [76–79].

**Figure 1:**
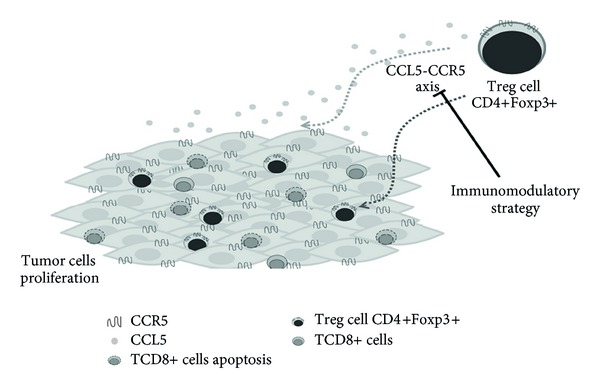
Schematic view of CCL5-CCR5 axis and Tregs involvement in tumor microenvironment. Tumors evade immune destruction by actively inducing immune tolerance through the recruitment of CD4+CD25+Foxp3+ regulatory T cells, which preferentially express CCR5 in the surface.

Cancer is a complex disease, where many alterations should be addressed in transformed cells. The CCR5 could have an influence on cancer formation, progression, and prognosis, but this influence is not so sharp, in spite of recent results that have clarified many roles. Maybe in cancer development, Treg migration into the tumor microenvironment is mediated by the CCL5-CCR5 axis (Figure 1). In this context, enhancing their ability to regulate antitumor CD8+ T cells and other immune effector cells, encouraging cellular proliferation of tumor cells. Thus, blockade of this pathway may represent a novel immunomodulatory strategy for the treatment of cancer.

Action such as chemokine receptor and ligands may depend on interaction with signaling molecules by immunological or nonimmunological cells, and this may be modified by genetic changes, mRNA, and protein expression level. With a deeper understanding of the role of CCR5 in tumorigenesis process, it could be used as a molecular marker in diagnoses and prognoses and even in treatment of cancer.
